# Intercropping kura clover with prairie cordgrass mitigates soil greenhouse gas fluxes

**DOI:** 10.1038/s41598-020-64182-2

**Published:** 2020-04-30

**Authors:** Gandura Omar Abagandura, Udayakumar Sekaran, Shikha Singh, Jasdeep Singh, Mostafa A. Ibrahim, Senthil Subramanian, Vance N. Owens, Sandeep Kumar

**Affiliations:** 1 Department of Agronomy, Horticulture and Plant Science, South Dakota State University, Brookings, South Dakota USA; 2 Department of Biosystems Engineering and Soil Science, University of Tennessee, Knoxville, Tennessee USA; 3 Research & Development Department, Acatgro LLC, Fresno California, USA

**Keywords:** Climate-change mitigation, Environmental impact

## Abstract

Prairie cordgrass (PCG) (*Spartina pectinata* Link) has a high tolerance to soil salinity and waterlogging, therefore, it can thrive on marginal lands. Optimizing the nitrogen (N) input is crucial to achieving desirable biomass production of PCG without negatively impacting the environment. Thus, this study was based on the hypothesis that the use of legumes such as kura clover (*Trifolium ambiguum* M. Bieb.) (KC) as an intercrop with PCG can provide extra N to the crop reducing the additional N fertilizer and mitigating soil surface greenhouse gas (GHG) emissions. Specific objective of the study was to assess the impact of PCG managed with different N rates [0 kg N ha^−1^ (PCG-0N), 75 kg N ha^−1^ (PCG-75N), 150 kg N ha^−1^ (PCG-150N), and 225 kg N ha^−1^ (PCG-255N)], and PCG intercropped with KC (PCG-KC) on GHG fluxes and biomass yield. The experimental site was established in 2010 in South Dakota under a marginally yielding cropland. The GHG fluxes were measured from 2014 through 2018 growing seasons using the static chamber. Net global warming potential (GWP) was calculated. Data showed that cumulative CH_4_ and CO_2_ fluxes were similar for all the treatments over the study period. However, the PCG-KC, PCG-0N, and PCG-75N recorded lower cumulative N_2_O fluxes (384, 402, and 499 g N ha^−1^, respectively) than the PCG-150N (644 g N ha^−1^) and PCG-255N (697 g N ha^−1^). The PCG-KC produced 85% and 39% higher yield than the PCG-0N in 2016 and 2017, respectively, and similar yield to the other treatments (PCG-75N, PCG-150N, and PCG-255N) in these years. Net GWP was 52% lower for the PCG-KC (112.38 kg CO_2_-eq ha^−1^) compared to the PCG-225N (227.78 kg CO_2_-eq ha^−1^), but similar to other treatments. Soil total N was 15%% and 13% higher under PCG-KC (3.7 g kg^−1^) than that under PCG-0N (3.2 g kg^−1^) and PCG-75N (3.3 g kg^−1^), respectively. This study concludes that intercropping prairie cordgrass with kura clover can enhance biomass yield and reduce fertilizer-derived N_2_O emissions and net global warming potential.

## Introduction

Carbon dioxide, methane, and nitrous oxide, the primary greenhouse gases (GHGs), have drawn a lot of attention from scientists and the public worldwide due to their important role in the global warming potential^1^. One of the main causes of GHG emissions in the United States (US) is the agricultural practices^2^. The negative effects of GHG emissions have promoted research in the sustainable agricultural systems that can mitigate these emissions^3,4^. Perennial grasses can replace fossil fuel energy sources and decrease GHG emissions through enhancing soil organic matter^5^. Prairie cordgrass (PCG) (*Spartina pectinata* Link.), a native perennial grass mostly found in Northeast and Midwest of the US and Canada may be used as a source for biofuel production^6^. Prairie cordgrass can grow under high soil salinity levels and flooded conditions; therefore, it can thrive on marginal lands where other crops are not adapted^7^. The marginal lands are considered one of the main resources that can provide biofuel production, thus reducing land-use competition between energy and food crops^8^.

Nitrogen (N) management is essential for optimizing the biomass yield of prairie cordgrass^9,10^. However, inappropriate fertilizer inputs can increase microbial activity and root respiration, which in turn can increase GHG emissions^4^. Therefore, the lower rates of fertilizer application in PCG could potentially help in reducing the GHG emissions associated with energy production. Intercropping with legumes as a source of N in bioenergy cropping systems has also been widely used to reduce N fertilizer application^11^. In nonlegume-legume mixture, rhizobia living in the root nodules of legumes can fix atmospheric N, which subsequently can be transferred to nonlegume crop through the decomposition of legume residues and roots^12^. Several studies have reported the effect of intercropping bioenergy crops with legumes on GHG emissions. For example, a study by^13^ reported that intercropping white clover (*Trifolium repens* L. cv. Klondike) with perennial ryegrass (*Lolium perenne* L. cv. Fanda) did not impact nitrous oxide (N_2_O) emissions compared to perennial ryegrass only. Epie *et al*.^14^ found the N_2_O cumulative emissions of a mixture of reed canarygrass (*Phalaris arundinacea*) and galega (*Galega orientalis*) were lower than those from reed canarygrass soils, which was attributed to the fact that N is fixed symbiotically within the legume nodules and thus is not freely available in the soil. However, legumes in bioenergy production may increase N_2_O emission by (1) supplying the microbial community in the soil with N compounds, and (2) the decomposition of residues from legume plants^15^. Intercropping corn (*Zea mays* L.) with either soybean (*Glycine max* L. [Merr.])^16^ or pea *(Pisum sativum*)^17^ reduced carbon dioxide (CO_2_) emissions compared to the corn alone. Shen *et al*.^18^ reported that intercropping corn with soybean did not affect methane (CH_4_) emissions compared to corn alone. Carbon dioxide and CH_4_ emissions can be mitigated by increasing the amount of C stored in soils^19^. Legumes can deliver more of the N needed to store C into the soil^20^; therefore, it may be one of the ways to mitigate these gases from the bioenergy cropping system.

Kura clover (KC) (*Trifolium ambiguum* M. Bieb.) is a perennial legume that can be grown in combination with perennial grasses to improve yields by providing biologically fixed N^7^. Due to its N-rich biomass and high tolerance to drought, flood, and cold, the effect of intercropping KC on yield has received a lot of attention^21,22^. According to Zemenchik *et al*.^23^, intercropping KC with cool-season forage grasses replaced between 74 and 336 kg N ha^–1^ of N fertilizer requirements. The effects of intercropping KC with PCG on soil GHG emissions have not been evaluated. Because N would be fixed symbiotically within the KC nodules in the PCG-KC mixture, and thus is not freely available in the soil compared to N fertilizer additions, we hypothesized that intercropping KC with PCG in the marginal lands would improve the yield of PCG and reduce soil GHG emissions compared to unfertilized and fertilized PCG. The objectives of this study were to 1) study how different N rates (0, 75, 150, and 225 kg N ha^−1^) can affect GHG emissions (CO_2_, N_2_O and CH_4_) from PCG, and 2) determine the effect of intercropping KC with PCG on GHG emissions and PCG yield compared to unfertilized and fertilized PCG.

## Results

### Soil temperature and water content

**Figure 1:**
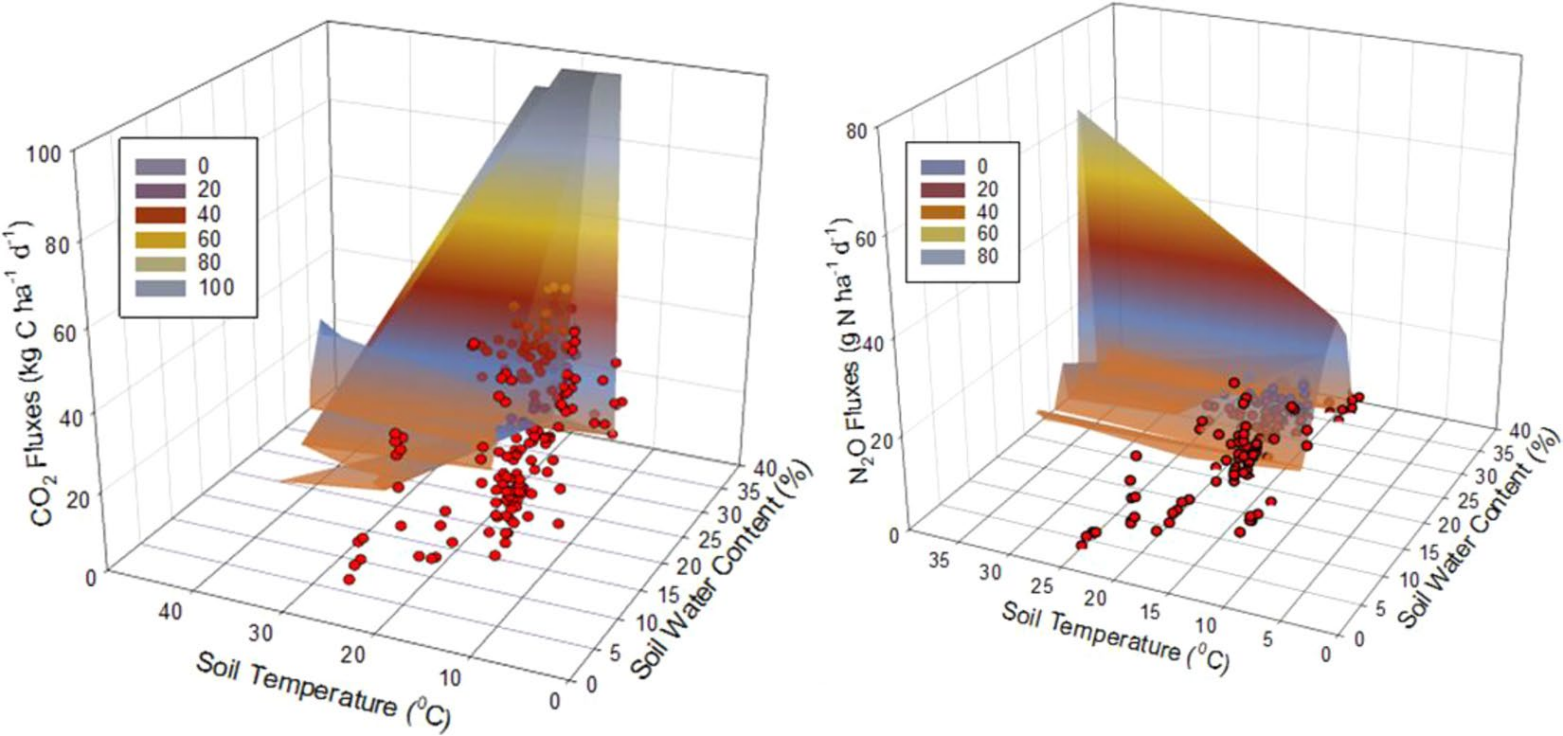
Correlation of the combination of soil temperature and soil water content with daily CO_2_ and N_2_O fluxes for 2014–2018 growing seasons.

Soil temperature and soil water content during the study period (data not shown) increased with increasing air temperature and precipitation, respectively. Soil water content, regardless of management, was significantly affected among years (*P* = 0.0341), with 2014 and 2015 growing seasons having lower soil water content than the 2016 and 2018 seasons. However, mean soil temperature and water content in each year were similar (*P values* > 0.05 for each year and each soil parameter) under all the treatments (N fertilization rates and intercropping of KC). Soil water content and temperature were significantly correlated with CO_2_ (*P* = 0.0039) and N_2_O (*P* = 0.0189) fluxes, with the combination of these two soil parameters explaining 24% and 27% of the fluxes of CO_2_ and N_2_O variation, respectively, (Fig. 1).

### Soil properties

**Figure 2:**
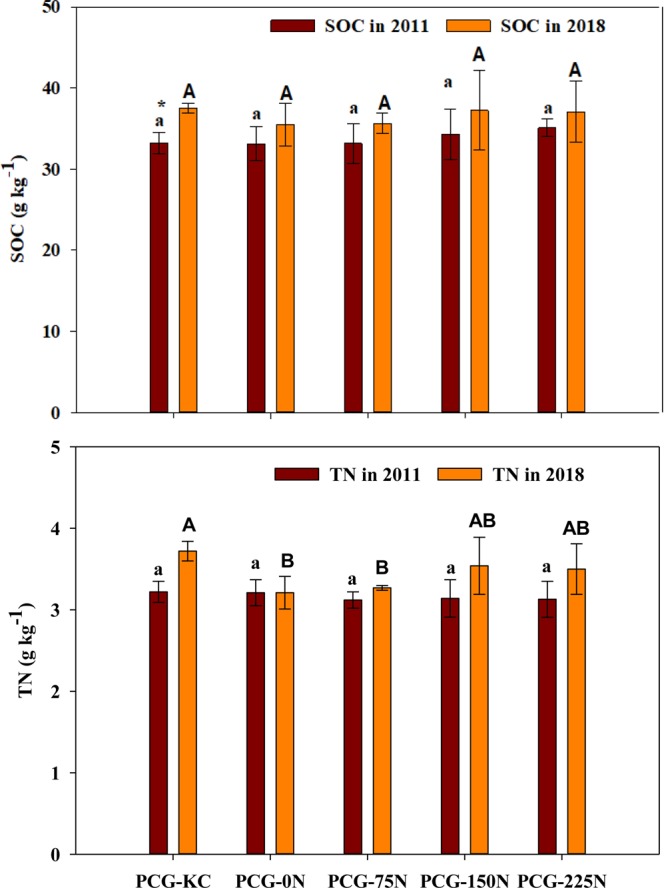
Soil organic carbon (SOC, g kg^-1^) and total N (TN, g kg^-1^) in 2011 and 2018 at 0–15 cm depth as influenced by the mixture of prairie cordgrass and kura clover (PCG-KC) and prairie cordgrass managed with different nitrogen rates (0 kg N ha^-1^ (PCG-0N), 75 kg N ha^−1^ (PCG-75N), 150 kg N ha^−1^ (PCG-150N) and 225 kg N ha^−1^ (PCG-225N)). Vertical bars indicate standard errors of the means (n = 4). Different small letters indicate a significant difference among treatments in 2011 and different capital letters indicate a significant difference among treatments in 2018. (*) indicates a significant difference between the two years in each treatment.

Soil pH ranged from 8.0 to 8.4 and EC from 0.33 to 0.35 dS m^−1^ in 2018. Nitrogen fertilization rates and intercropping of KC did not affect pH and EC (*P* values ≤ 0.05). Soil organic C (SOC) and total N (TN) in 2011 and 2018 are presented in Fig. 2. The range of SOC was from 30.9 to 37.1 g kg^−1^ in both years. Nitrogen fertilization rates and intercropping of KC did not show any significant impact on SOC in both years (Fig. 2). However, SOC did not change within the N fertilization rates over time; yet, it was 13.5% higher in 2018 than in 2011 under PCG-KC plots. Total N ranged from 2.9 to 4.0 g kg^−1^ in both years. Total N content over time (from 2011 to 2018) did not significantly change for each treatment; however, N fertilization rates and intercropping of KC in 2018 significantly affected TN (*P* = 0.0314). In 2018, PCG-KC recorded significantly 15% and 13% higher soil TN (3.7 g kg^−1^) than PCG-0N (3.2 g kg^−1^) and PCG-75N (3.3 g kg^−1^), respectively, but similar TN to PCG-150N (3.5 g kg^−1^) and PCG-225N (3.6 g kg^−1^).

### Biomass yield

Biomass yields of PCG in 2015, 2016 and 2018 are listed in Table 1. Overall, biomass production ranged from 4.3 to 7.3 Mg ha^−1^ in 2015, 4.1 to 15.1 Mg ha^−1^ in 2016, and 5.3 to 13.9 Mg ha^−1^ in 2018. In 2015, no significant differences on yield among treatments were observed; however, the yield was significantly affected by N fertilization rates and intercropping of KC in 2016 and 2018 (Table 1). In 2016, PCG-KC recorded 85% higher yield (11.19 Mg ha^−1^) than PCG-0N (6.05 Mg ha^−1^), but similar yield as PCG-75N (9.86 Mg ha^−1^), PCG-150N (10.88 Mg ha^−1^), and PCG-225N (14.08 Mg ha^−1^). Similar trends were also observed in 2018, with PCG-KC recording 39% higher yield (10.41 Mg ha^−1^) than PCG-0N (7.5 Mg ha^−1^), but similar yield as PCG-75N (8.39 Mg ha^−1^), PCG-150N (10.31 Mg ha^−1^), and PCG-225N (12.24 Mg ha^−1^).

**Table 1:** Yield (Mg ha^−1^) for 2015, 2016 and 2018 of the mixture of prairie cordgrass and kura clover (PCG-KC), prairie cordgrass managed with different nitrogen rates (0 kg N ha^−1^ (PCG-0N), 75 kg N ha^−1^ (PCG-75N), 150 kg N ha^−1^ (PCG-150N), and 225 kg N ha^−1^ (PCG-225N)). Standard errors values (±) are shown in the parentheses.

	Biomass yield (Mg ha^−1^)		
	2015	2016	2018
PCG-KC	6.60 (±3.2)^†^	11.19 (±3.6) ab	10.41(±0.6) ab
PCG-0N	6.14 (±2.7)	6.05 (±2.1) c	7.50 (±2.2) c
PCG-75N	6.48 (±3.9)	9.86 (±2.2) bc	8.39 (±1.2) bc
PCG-150N	6.38 (±1.6)	10.88 (±2.0) ab	10.31 (±4.1) ab
PCG-225N	5.88 (±2.0)	14.08 (±3.0) a	12.24 (±1.4) a
	**Analysis of variance (P** > **F)**		
	0.8321	0.0411	0.0381

^†^Within columns, means followed by different lower-case letter are significantly different (*P* = 0.05).

### Daily and cumulative CO_2_, CH_4_ and N_2_O fluxes

**Figure 3:**
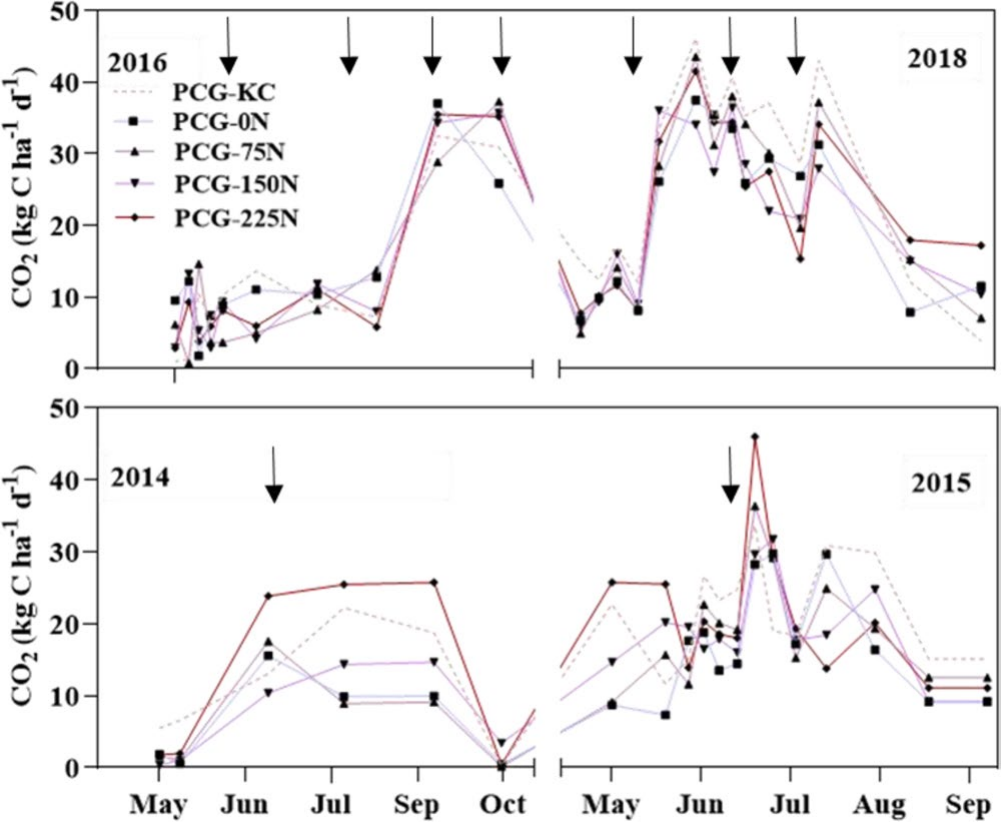
Trends of CO_2_ fluxes over growing seasons of 2014, 2015, 2016 and 2018 as influenced by the mixture of prairie cordgrass and kura clover (PCG-KC), and prairie cordgrass managed with different nitrogen rates (0 kg N ha^−1^ (PCG-0N), 75 kg N ha^−1^ (PCG-75N), 150 kg N ha^−1^ (PCG-150N) and 225 kg N ha^−1^ (PCG-225N)). The black bold arrows indicate heavy precipitation events.

Mean CO_2_ fluxes at each sampling date in 2014, 2015, 2016, and 2018 growing seasons under N fertilization rates and KC intercropping are shown in Fig. 3. As it was expected, only positive values of CO_2_ fluxes were observed under all treatments in all years. Generally, N fertilization rates and KC intercropping emitted higher CO_2_ flux at the middle and end of the growing seasons compared to the beginning of the growing seasons, with peaks of this gas being observed in all years. Carbon dioxide fluxes under N fertilization rates and KC intercropping generally showed a similar pattern in 2016 and 2018. However, the PCG-225N seemed to emit higher CO_2_ fluxes than all the other treatments at most dates in 2014 and 2015, but no significant differences on daily CO_2_ fluxes were recorded between PCG-225N and other treatments at all dates (*P* ≤ 0.05 for all years). The cumulative CO_2_ fluxes under N fertilization rates and KC intercropping are listed in Table 2. Differences on cumulative CO_2_ fluxes among treatments were not significant, with PCG-KC, PCG-0N, PCG-75N, PCG-125N, and PCG-225N recording cumulative CO_2_ fluxes of 2731.47 kg ha^−1^, 2053.09 kg ha^−1^, 2170.36 kg ha^−1^, 2337.64 kg ha^−1^, and 2661.64 kg ha^−1^, respectively, (Table 2).

**Table 2:** Cumulative soil surface CO_2_, CH_4_ and N_2_O fluxes for the mixture of prairie cordgrass and kura clover (PCG-KC), and prairie cordgrass managed with different nitrogen rates (0 kg N ha^−1^ (PCG-0N), 75 kg N ha^−1^ (PCG-75N), 150 kg N ha^−1^ (PCG-150N) and 225 kg N ha^−1^ (PCG-225)). Standard errors values (±) are shown in the parentheses.

	Cumulative GHG fluxes		
	CO_2_ flux(kg C ha^−1^)	CH_4_ flux(g C ha^−1^)	N_2_O flux(g N ha^−1^)
PCG-KC	2731.47 (±66.6)^†^	348.62 (±46.4)	384.15 (±58.7) b
PCG-0N	2053.09 (±42.1)	342.24 (±55.8)	402.21 (±79.4) b
PCG-75N	2170.36 (±34.5)	226.71 (±49.8)	499.16 (±68.9) b
PCG-150N	2337.64 (±161.1)	244.83 (±44.4)	644.55 (±89.7) a
PCG-225N	2661.64 (±222.4)	355.95 (±50.2)	697.24 (±51.8) a
	**Analysis of variance (P** > **F)**		
Treatments	0.4321	0.3276	0.0034

†Within columns, means followed by different letter(s) are significantly different (P = 0.05).

**Figure 4:**
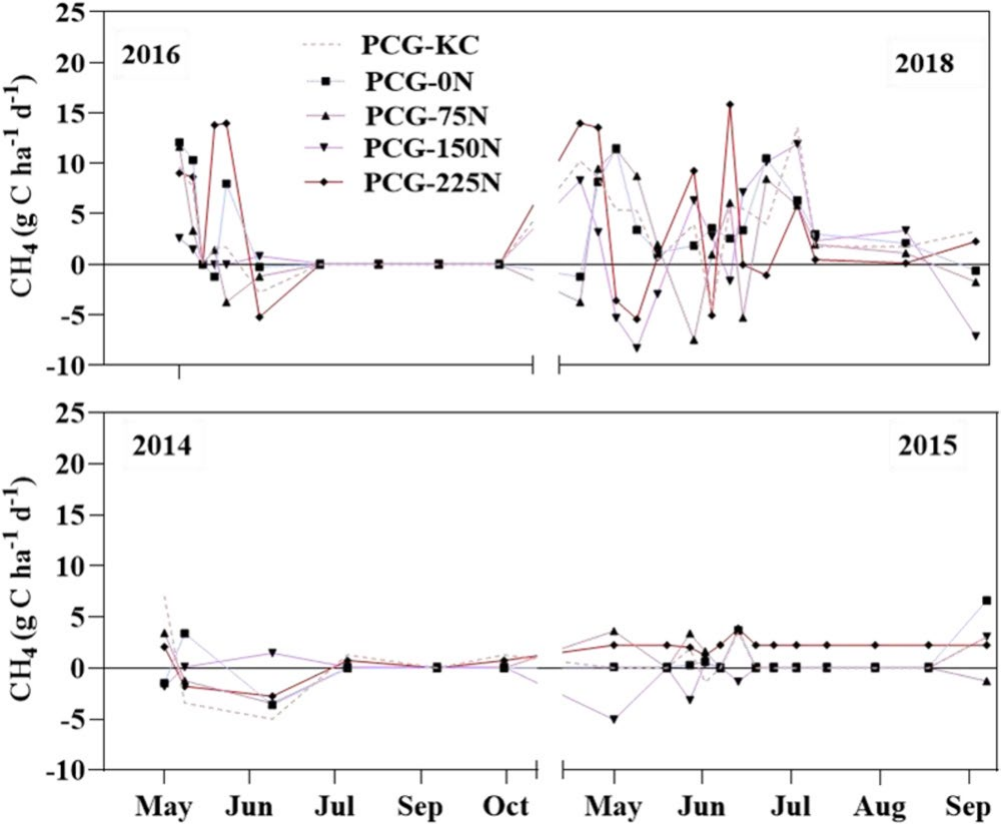
Trends of CH_4_ fluxes over growing seasons as influenced the mixture of prairie cordgrass and kura clover (PCG-KC), and prairie cordgrass managed with different nitrogen rates (0 kg N ha-1 (PCG-0N), 75 kg N ha^−1^ (PCG-75N), 150 kg N ha^−1^ (PCG-150N) and 225 kg N ha^−1^ (PCG-225N)).

Daily trend of CH_4_ under N fertilization rates and KC intercropping in the four years are shown in Fig. 4. Methane flux was different from the CO_2_ flux, where both negative and positive CH_4_ fluxes were observed in all growing seasons in this study. Methane fluxes were lower in 2014 and 2015 seasons compared to 2016 and 2018 seasons, with high positive and negative values of CH_4_ fluxes being observed in the two latter seasons. Daily CH_4_ fluxes were not affected by N fertilization rates and KC intercropping in all years (*P* values ≤ 0.05 for all years). Table 2 listed the cumulative CH_4_ fluxes under N fertilization rates and intercropping of KC. Similar cumulative CH_4_ fluxes were recorded under N fertilization rates and KC intercropping (Table 2).

**Figure 5:**
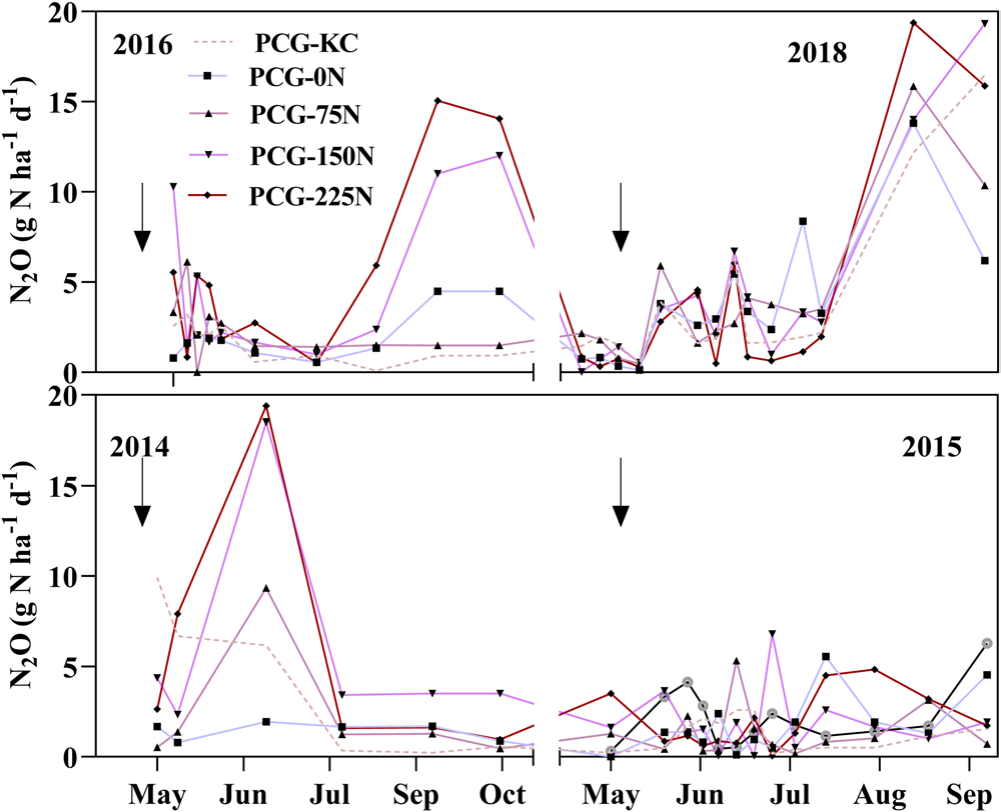
Trends of N_2_O fluxes over growing seasons of 2014, 2015, 2016 and 2018 as influenced by the mixture of prairie cordgrass and kura clover (PCG-KC), and prairie cordgrass managed with different nitrogen rates (0 kg N ha^−1^ (PCG-0N), 75 kg N ha^−1^ (PCG-75N), 150 kg N ha^−1^ (PCG-150N) and 225 kg N ha^−1^ (PCG-225N)). The black bold arrows indicate when N fertilizer was applied.

Mean N_2_O fluxes at each sampling date under N fertilization rates and KC intercropping in the four years are shown in Fig. 5. Fluxes of N_2_O were different under N fertilization rates and KC intercropping; however, the trend of N_2_O flux under each treatment was generally similar in each year. Nitrogen fertilization rates and KC intercropping affected the daily N_2_O fluxes during 2014 and 2016 peaks, with PCG-225N and PCG-150N treatments recording higher N_2_O flux than other treatments (*P* values <0.05). The cumulative N_2_O fluxes under N fertilization rates and intercropping of KC are shown in Table 2. The PCG-KC (384.15 g ha^−1^) recorded 40% and 45% lower cumulative N_2_O fluxes than PCG-150N (644.55 g ha^−1^) and PCG-225N (697.24 g ha^−1^), respectively, but similar cumulative N_2_O fluxes as PCG-0N (402.21 g ha^−1^), and PCG-75N (499.16 g ha^−1^) (Table 2).

### Global warming potential

Global warming potential (GWP) for CH_4_ (GWP_CH4_) and N_2_O (GWP_N2O_) and net GWP can be seen in Table 3. Similar to cumulative CH_4_ flux, GWP_CH4_ was similar under N fertilization rates and KC intercropping. However, N rates and KC intercropping affected GWP_N2O_, with PCG-KC (104.37 kg CO_2_-eq ha^−1^) recording 52% lower GWP_N2O_ than PCG-225N (219.59 kg CO_2_-eq ha^−1^), but similar GWP_N2O_ as other treatments (Table 3). The inputs of GWP_N2O_ in the net GWP was higher than the inputs of GWP_CH4_ over the study period. Similar to GWP_N2O,_ the net GWP under PCG-KC (112.38 kg CO_2_-eq ha^−1^) was 52% lower than under PCG-225N (227.78 kg CO_2_-eq ha^−1^), but similar to other treatments.

**Table 3:** Global warming potential for CH_4_ (GWP_CH4_), N_2_O (GWP_N2O_) and net global warming potential (net GWP) for the mixture of prairie cordgrass and kura clover (PCG-KC), and prairie cordgrass managed with different nitrogen rates (0 kg N ha^−1^ (PCG-0N), 75 kg N ha^−1^ (PCG-75N), 150 kg N ha^−1^ (PCG-150N) and 225 kg N ha^−1^ (PCG-225)). Standard errors values (±) are shown in the parentheses.

	Global warming potential		
	GWP_CH4_	GWP_N2O_	Net GWP
	(kg CO_2_-eq ha^−1^)		
PCG-KC	8.02 (±3.4)^†^	104.37 (±70.7) b	112.39 (±80.5) b
PCG-0N	7.87 (±3.2)	111.79 (±51.2) b	119.66 (±62.5) b
PCG-75N	5.21 (±1.3)	142.80 (±44.2) ab	148.02 (±70.2) ab
PCG-150N	5.63 (±2.4)	178.46 (±56.1) ab	184.09 (±40.7) ab
PCG-225N	8.19 (±3.2)	219.59 (±61.5) a	227.78 (±90.2) a
	**Analysis of variance (P** > **F)**		
Treatments	0.4213	0.0311	0.0127

^†^Within columns, means followed by different letter(s) are significantly different (P = 0.05).

## Discussion

### Effects of N rates and kura clover intercropping on soil water content and temperature

This study showed that soil water content and temperature were similar under all treatments (N rates and KC intercropping). Li *et al*.^4^ reported that N inputs ranged from 0 to 84 kg N ha^−1^ did not affect soil water content and soil temperature when applied to other bioenergy crops like camelina (*Camelina sativa* L.) and carinata (*Brassica carinata*). Intercropping reed canarygrass with galega did not affect soil water content as compared to reed canarygrass plots as reported by Epie *et al*.^14^. Nitrogen fertilizer additions to switchgrass (*Panicum virgatum* L.) at a rate of 112 kg ha^−1^ showed similar soil water content and temperature compared to none-fertilized soils^24,25^. Soil organic C was not affected by N rates and KC intercropping in 2018 (Fig. 2), perhaps explaining why these soil parameters were not affected in this study. Increased SOC can improve soil porosity, which in turn can increase soil water holding capacity^26^.

### Soil properties as influenced by N rates and kura clover intercropping

Nitrogen fertilizers can release hydrogen ions during nitrification process^27^, and legumes can acquire N as diatomic N rather than as nitrate^28^, signifying that both management practices can lower the soil pH. In addition, higher rates of fertilizer contain high salt concentrations, which may induce soil salnization^29^. However, N rates and KC intercropping with PCG had similar soil pH and EC in this study. Eight years of N fertilization and KC intercropping may not be enough to cause changes in soil pH and EC. Application of N fertilizer (0–112 kg N ha^−1^) to switchgrass for four years did not affect soil pH compared to the control in South Dakota^24^. Kidd *et al*.^30^ reported that N fertilizer applications for 120 years acidified the soil in the grassland ecosystem. Intercropping corn with bambara groundnut (*Vigna subterranea* (L) Verdc) for five years^31^ and wheat (*Triticum aestivum*) with faba beans (*Vicia faba* L.) for nine years^32^ did not affect soil pH compared to the sole cropping system. Liebig *et al*.^33^ reported that N fertilizer applications for 16 years affected soil salinization in the western corn belt.

Similar to pH and EC, treatments did not affect SOC in this study. High N fertilization rates^4^ and intercropping with legumes^34^ in bioenergy system increased biomass production resulting in an increase in SOC. The increase in biomass with higher N rate and PCG-KC mixture was observed in 2016 and 2018 (Table 1); however, this increased biomass was not translated into an increase in the SOC. The aboveground biomass was harvested in all years, which may caused the lack of effects of N fertilization rates and KC intercropping on SOC. Alvarez *et al*.^35^ reported that N application could increase the SOC only when crop residues were not removed. However, intercropping PCG with KC increased soil C over time, which may be attributed to greater root inputs than other N fertilization rates. Cong *et al*.^34^ conducted a study in China for seven years in a field experiment that compared corn grown as a sole crop with corn and faba beans growing as an intercropping system and reported that C sequestration rate was significantly higher in intercrop than in the sole crop. Higher TN under higher N rate and PCG-KC mixture in this study were attributed to N availability by the former^36^ and through N-fixation by the latter^37^. This increase in SOC over time and TN content under KC intercropping in this study emphases the fact that intercropping KC with PCG may be one of the effective ways to improve marginal land soils.

### Effects of N rates and kura clover intercropping on biomass yield

Higher biomass under higher N fertilization rates and KC intercropping was probably a result of increased N availability from these two management systems. This suggests that intercropping KC with PCG increased the N availability to PCG through N fixation^12^ and resulting in increased grass biomass yield. The PCG-KC had higher soil TN than the PCG-0N which can confirm the N availability to PCG through kura clover intercropping. These results concur with the findings of Gulwa *et al*.^38^, who reported that a mixture of red clover (*Trifolium pretense*) and native grasses in South Africa produced more yield in comparison with the grass only. Similarly, Lee *et al*.^39^ reported that 120 kg N ha^−1^ increased the biomass of *Miscanthus* × *giganteus* in Illinois compared to the none fertilized soils. This improvement in PCG biomass due to its intercropping with KC can provide more feedstock for the bioenergy from marginal lands, which in turn can help in lowering our dependency on fossil fuels.

### Trends and cumulative GHG fluxes as affected by N rates and kura clover intercropping

Higher N fertilizer rate and KC intercropping, which increased biomass yield in 2016 and 2018 (Table 1), were expected to promote daily CO_2_ fluxes during the active crop growth due to higher root and microbial respiration compared to lower N fertilizers rate; however, no increase in the daily CO_2_ fluxes due to these two managements were observed in these two years. Carbon dioxide fluxes varied from one year to another in this study, which may have caused the lack effect of N fertilization rates and KC intercropping on the cumulative CO_2_ fluxes. Other researchers also reported that N fertilization rates and intercropping of legumes did not affect cumulative CO_2_ fluxes. For example, Nikièma *et al*.^25^ and Li *et al*.^4^ reported that N fertilization rates did not affect CO_2_ emission compared to the unfertilized soil in switchgrass, camelina and carinata. Shen *et al*.^18^ reported that intercropping corn with soybean in China did not affect CO_2_ emissions compared to the corn alone. This non significant impact due to treatments on cumulative CO_2_ fluxes was also attributed to the absence effects of N fertilizer rate and kura clover intercropping on the measured soil properties (pH, EC and SOC). For example, Abagandura *et al*.^40^ reported that microbial activity which plays a major role in decomposing soil organic matter and CO_2_ emissions could be affected by soil pH. Increasing soil EC due to management practices can decrease microbial activity resulting in a reduction in soil respiration^41^. The change in SOC can influence soil respiration as reported by Jin *et al*.^42^. In addition, soil temperature and water content were similar under all N fertilizer rates and intercropping KC, which may cause similar CO_2_ flux from these managements in this study^43^.

The experimental field received lower precipitation in 2014 and 2015 compared to 2016 and 2018 (Table 4), which may have caused lower CH_4_ in the former than latter^40^. Higher soil water content can increase CH_4_ emission as reported by Mbonimpa *et al*.^24^. Watts *et al*.^44^ reported lower soil CH_4_ fluxes under aerobic soil conditions. Similar soil water content in this reported study may have resulted in similar cumulative CH_4_ fluxes among treatments. A study by^4^ also reported no significant effect of N fertilizer rates on soil CH_4_ fluxes compared with the unfertilized soils in the dryland cropping system. Shen *et al*.^18^ reported that intercropping corn with soybean in China did not affect CH_4_ fluxes compared to corn alone. However, Sainju *et al*.^19^ reported that CH_4_ flux was reduced with N fertilizer and legumes, which was attributed to greater root growth under these two managements, which can result in CH_4_ absorption.

**Table 4:** Total monthly precipitation, maximum and minimum air temperature in 2014, 2015, 2016, and 2018 growing seasons at the study site.

Year	Apr.	May	Jun.	Jul.	Aug.	Sep.	Oct.	Total
**Precipitation (mm)**								
2014	3.48	50.00	3.70	0.18	1.42	54.23	8.80	121.81
2015	4.48	0.00	1.23	101.18	0.01	0.96	2.80	110.66
2016	23.86	100.92	0.57	112.2	100.01	99.99	22.24	459.79
2018	8.29	0.55	200.44	102.65	0.19	0.84	0.00	312.96
**Maximum air temperature (**°C**)**								
2014	16.36	19.04	21.82	18.86	20.32	22.33	19.52	
2015	16.43	16.34	21.33	23.03	21.60	21.51	20.08	
2016	16.33	19.64	19.61	20.49	19.09	21.68	15.81	
2018	14.94	17.31	20.42	20.82	18.39	20.64	17.81	
**Minimum air temperature (**°**C)**								
2014	7.05	9.95	12.35	12.07	11.84	13.32	12.30	
2015	6.60	9.20	11.56	14.03	13.30	11.82	11.82	
2016	8.88	10.31	10.73	11.44	10.44	11.06	10.69	
2018	7.34	9.73	10.40	11.98	11.66	10.88	10.01	

Increased cumulative N_2_O fluxes under higher N fertilizer rates compared to lower and unfertilized PCG, and PCG-KC was attributed to increasing in the N availability substrate under higher rates enahncing soil microbes activity, thereby increasing N_2_O flux^25,45^. Intercropping KC with PCG decreased cumulative N_2_O flux compared to the higher N fertilizer rates, probably due to N fixation within the KC nodules in the PCG-KC and thus is not freely available in the soil compared to higher N addition to PCG. These finding supported the results reported by Senbayram *et al*.^46^, who found that intercropping wheat with faba bean reduced cumulative N_2_O emissions compared with N-fertilized wheat. In addition, higher particulate N was found in PCG-225N and PCG-125N than in PCG-KC, PCG-0N and PCG-75N in 2018 (data not shown). This labile N can increase mineralization through enhancement of microbial activity, thus resulting in an increase in N_2_O flux^47^.

### Effects of N rates and kura clover intercropping on global warming potential

The GWP_CH4_ was similar under N rates and KC intercropping, which can be attributed to the lack effect of these treatments on CH_4_ flux. Higher GWP_N2O_ under higher N rate in this study was attributed to the availability of N substrate under higher N rates increasing microbial N_2_O production^45^ as discussed above. Because fluxes of CH_4_ was small in this study, the major part of net GWP resulted from GWP_N2O._ Dryland cropping system always emits a small quantity of CH_4_^33,35^. Higher GWP_N2O_ under higher N rates in this study had resulted in higher net GWP from these treatments compared to PCG-KC. In summary, intercropping KC with PCG decreased N_2_O flux and resulting net GWP. This finding concludes that such management may decrease the concern of GHG emissions associated with bioenergy crops.

### Management implementation potential and its implication on the environment

Because fossil fuels are non-renewable and negatively impact the environment, the interest of using sustainable sources grown on marginal lands has increased worldwide. The total area of marginal land in the US varies significantly from one study to another^48^. According to Boe *et al*.^7^, there is more than 210,000 ha in South Dakota that are classified as wetlands and are not suitable for conventional crop production. Prairie cordgrass, which is mostly found in flooded soils in the Northeast and Midwest of the US, could potentially be used as a biofuel feedstock on the marginal lands. One of the challenges in prairie cordgrass cropping systems is N fertilizer management. Although it is accepted that legumes can provide N to the soil, there is no study evaluating how intercropping a legume like kura clover with prairie cordgrass in marginal lands can affect prairie cordgrass yield and GHG emissions. This is the first study evaluating how intercropping kura clover with prairie cordgrass can affect grass biomass, soil properties, GHG emissions and GWP. Results from this study suggested that intercropping kura clover with prairie cordgrass increased total N, SOC over time, increased the yield of prairie cordgrass, and mitigated GHG emissions from marginal land cropping systems. Kura clover and prairie cordgrass intercropping in marginal lands not only improves soil and allows such lands to be productive; it also decreases the concern from bioenergy regarding the use of edible crops and GHG emissions. Considering the environmental damage from fossil fuels and N fertilizers, utilizing prairie cordgrass/kura clover mixtures for bioenergy may mitigate GHG emissions by reducing N fertilizer requirements and improve soil and environment quality.

## Conclusion

This study evaluated the response of soil surface GHG fluxes and resulting GWP and prairie cordgrass to N fertilization rates and intercropping kura clover with prairie cordgrass. Soil data for 2011 and 2018 showed that N fertilization rates and intercropping K did not affect soil pH, EC and SOC. However, PCG-KC increased total N and stored more C compared to the lower and unfertilized PCG in 2018. The PCG-KC produced a higher yield than the unfertilized PCG, and was similar to the fertilized PCG in 2016 and 2018. The cumulative CO_2_ and CH_4_ fluxes for 4 years (2014, 2015, 2016 and 2018) were not influenced by the application of N fertilization rates and PCG-KC. However, the cumulative N_2_O fluxes and net GWP for 4 years increased under the higher N fertilization rate compared to the PCG-KC. In general, this study suggests that intercropping kura clover with prairie cordgrass has a good potential for mitigating N_2_O emissions and global warming potential.

## Materials and Methods

### Site description and treatment details

The study was initiated in 2010 and conducted in 2014, 2015, 2016, and 2018. The study was also conducted in 2017; however, all gathered data in this year was lost. This field experiment was located in Felt Research Farm at South Dakota State University in Brookings, SD, US (44° 22′ N and 96° 47′ W). The soil of the site was Orthic Anthrosol^49^. The soil is poorly drained or commonly flooded for some period during spring. Soil pH and EC were 8.31 and 0.38 dS m^−1^, respectively. The experimental site generally has hot summers and freezing winters. From 2010 to 2018, mean annual precipitation was 466 mm and mean annual temperature was 5.9 °C. The study included four N fertilizer treatments and one intercrop treatment: PCG with 0, 75, 150, and 225 kg ha^−1^ (PCG-0N, PCG-75N, PCG-150N, and PCG-225N, respectively) and PCG with KC (PCG-KC). The experimental design was a randomized complete block design with four replications. The granular urea (46% N) fertilizer was broadcasted manually in May each year (2010–2018)

Seeds of PCG and KC were obtained from a natural population in South Dakota and Ag Research New Zealand, respectively. Seedlings of PCG and KC were grown individually in containers (Stuewe, Inc, Corvallis, OR) during early spring 2010 in the greenhouse and transplanted in the field in later spring at the same year. The size of each field plot was 3.0 m wide and 5.7 m long. There have been nine rows in each plot. Kura clover seedling was transplanted on 30 cm centers within rows in the field. The density of K was 111,111 plants ha^−1^. After that, PCG seedlings (intercropping and monoculture) were transplanted in the field on 60 cm centers, with a density of 26,896 plants ha^−1^. In October 2015, 2016, and 2018, the yield was determined for all plots. From each plot, all biomass was harvested using a sickle-bar mower. To measure the grass fresh weight, all fresh harvested biomass was weighed, and then biomass subsamples from each plot were dried at 60 °C for 72 h to measure the grass dry weight.

### Soil analyses

Soil samples were taken in 2011 and in 2018 using a core with a diameter of 3.2-cm. Seven soil samples from each plot at a depth of 0–15 cm were taken from random spots. These soil samples were composited to represent one sample for each plot. Soil pH and electrical conductivity (EC) were measured using pH and EC meter. Dry combustion was used to measure SOC and TN. To remove inorganic C from SOC samples before combustion, the soils were pretreated with acid.

### Soil GHG sampling

Soil GHG fluxes were measured every week from May to October in 2014, 2015, 2016, and to 2018. Static chamber technique was used for GHG measurements. Details about the GHG measurements can be found in our previous papers^4,40^. Briefly, PVC chambers (one chamber in each plot) were installed to measure GHG fluxes. During GHG sampling, PVC lids were used to cover the installed chambers. To ensure no gases were released from the chambers; the lids closed the chambers firmly^50^. GHG sampling was measured at an interval time of 0, 20, and 40-minute using a syringe to extract these gases^28^. Gas samples were transferred to the lab, and GHG (CO_2_, CH_4_, and N_2_O) concentrations were analyzed using a gas chromatograph (GC-2014, Shimadzu Co., Ltd., Japan). Soil GHG fluxes were calculated as the change in headspace gas concentration over time within the enclosed chamber volume^42^. Cumulative fluxes were calculated using linear interpolation^40^. Global warming potential (GWP) in CO_2_-equivalent was estimated using radiative forcing of 296 and 23 for N_2_O and CH_4_, respectively, and because stock of SOC changes was not evaluated in this study, GWP for CO_2_ was not calculated^48^. The summation of GWP for CO_2_ and N_2_O (Net GWP) was also determined.

At the time of GHG measurements, soil water content and temperature were also measured. Climatic data (minimum and maximum air temperature and precipitation) in 2014, 2015, 2016, and 2018 growing seasons were obtained from Northeast Regional Climate Center^51^ and are shown in Table 4. Total precipitation in the 2016 growing season (459.7 mm) was 277% higher than in 2014 (121.8 mm), 315% higher than in 2015 (110.7 mm), and 47% higher than in 2018 (313.0 mm) (Table 4). The trend of air temperature was similar in all years, with the beginning of the growing season having higher air temperature than the middle and the end of the season (Table 4).

### Statistical analyses

Repeated measures analysis in PROC MIXED were used to analyze daily, and cumulative GHG fluxes, with treatments and replications, were considered as fixed and random variables, respectively. Sampling date in daily GHG fluxes and year in cumulative fluxes were considered as repeated measure variables. For treatment comparisons, Tukey’s test was used at 0.05 probability. Multiple regression (soil temperature and soil water content as explanatory variables and CO_2_ and N_2_O fluxes as dependent variables) was conducted in Sigma Plot 14.0 (Fig. 5).
